# Hybrid-based Bayesian algorithm and hydrologic indices for flash flood vulnerability assessment in coastal regions: machine learning, risk prediction, and environmental impact

**DOI:** 10.1007/s11356-022-19903-7

**Published:** 2022-03-29

**Authors:** Sherif Ahmed Abu El-Magd, Ali Maged, Hassan I. Farhat

**Affiliations:** Geology Department, Faculty of Science, Suez University, P.O. Box 43518, El Salam City, Suez Governorate Egypt

**Keywords:** Flash floods, Naïve Bayes, Machine learning algorithm, Vulnerability map, Hydrologic indices

## Abstract

Natural hazards and severe weather events are a matter of serious threat to humans, economic activities, and the environment. Flash floods are one of the extremely devastating natural events around the world. Consequently, the prediction and precise assessment of flash flood-prone areas are mandatory for any flood mitigation strategy. In this study, a new hybrid approach of machine learning (ML) algorithm and hydrologic indices opted to detect impacted and highly vulnerable areas. The obtained models were trained and validated using a total of 189 locations from Wadi Ghoweiba and surrounding area (case study). Various controlling factors including varied datasets such as stream transport index (STI), stream power index (SPI), lithological units, topographic wetness index (TWI), slope angle, stream density (SD), curvature, and slope aspect (SA) were utilized via hyper-parameter optimization setting to enhance the performance of the proposed model prediction. The hybrid machine learning (HML) model, developed by combining naïve Bayes (NïB) approach and hydrologic indices, was successfully implemented and utilized to investigate flash flood risk, sediment accumulation, and erosion predictions in the studied site. The synthesized new hybrid model demonstrated a model accuracy of 90.8% compared to 87.7% of NïB model, confirming the superior performance of the obtained model. Furthermore, the proposed model can be successfully employed in large-scale prediction applications.

## Introduction

Flash floods are considered as one of the most dangerous natural hazards causing extensive damages to property, and their impact extended to the ecosystem and humans. Communication and transportation systems, infrastructure, crops, social facilities, and service and educational buildings could also be affected by flash floods triggering a considerable economic loss. Based on the reported investigation by the World Meteorological Organization, the property loss due to flash floods was ranked in the top 10 among the other natural hazards in 75% of countries (Ashley and Ashley 2008). World widely in 2011 and 2012, the estimated economic loss during these two years owing to flood hazards was found to be €95 billion in addition to 200 million individuals affected by flash flood events (Ceola et al. 2014). Furthermore, the economic impact of flash floods and other natural hazards on developing countries is higher than the economically developed countries (Loayza et al. 2012). For instance, in the USA, a total number of 28,826 flash floods were reported during a period between 2007 and 2015, causing colossal property and crop damages in addition to more than 278 people lost their lives (Gourley et al. 2017).

Machine learning (ML) is part of algorithmic and heuristic approaches that are designed to understand correlations in specific datasets through intuitive training. Various researchers and scientists have reported ML approaches toward analysis and forecast studies for hydrology, floods, and landslides analysis and prediction (Abu El-Magd et al. 2021a, b, c; Al-Abadi 2018; Ali et al. 2013; Khosravi et al. 2019; Rahmati et al. 2019; Shahabi et al. 2020; Zhao et al. 2019). Flood predictions were performed with many ML techniques in order to evolve a flood management system. Many investigations and studies have been carried out on flood assessment and modeling using hydrological studies, physical modeling, GIS, and remote sensing (Pradhan et al. 2014; Liu et al. 2019; Abu El-Magd et al. 2021b). However, data-driven prediction and forecasting using ML models are promising tools as they are easier to apply with minimal inputs. ML models are popular due to the ability to numerically formulate the flood nonlinearity based on the historical dataset. Thus, ML algorithms have been steadily improving, demonstrating their ability for flood forecasting with a reasonable rate of outperforming traditional approaches. Furthermore, the ML was reported as an effective tool for prediction and forecasting including numerous studies integrating ML algorithms, e.g., artificial neural network (ANN), random forest (RF), extreme gradient boosting (XGB), boosted regression tree (BRT), K-nearest neighbor (KNN), and general linear model (GLM) (Sulaiman and Wahab 2017; Abu El-Magd et al. 2021b; Abu El-Magd 2022).

In the last decades, a large number of individual death events have been recorded due to flash floods in Egypt. In November 1994, a total of 600 individual death were recorded during 2–4 days of flooding that hit different areas in Egypt (Vries et al. 2013). Egypt was affected by a highly-rated flood in October 2019 that has not been experienced in the last 50 years. Generally, in Egypt, flooding began approximately in the middle of August and intensified in October, with considerably higher rainfall on the Ethiopian plateau (Negm and Omran 2020). Despite the great efforts for flood prediction and mitigation, more research and investigations are needed to improve the monitoring system. Investigation and analysis of flash floods in the Egyptian Eastern Desert (EED) were conducted by other researchers to evaluate the influence of its morphological parameters and associated risk (El Shamy 1992; Ghoneim et al. 2002; EL-Rayes et al. 2009; Abdalla et al. 2014; Abu El-Magd 2019).

**Fig. 1:**
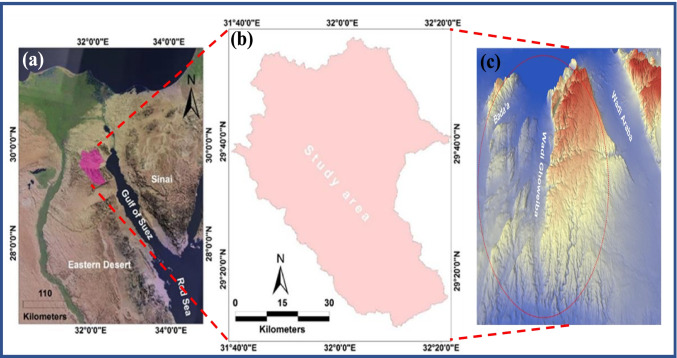
**a** Location map of the studied site in the Egyptian Eastern Desert, **b** the study area delineations, and **c** 3D map of the studied site

The study area (Wadi Ghoweiba and Wadi Bada’a) occupied the area between North El-Galala to the south and El-Sokhna Road to the north (Fig. 1). Ghoweiba basin and its tributaries mostly drained their water into the Gulf of Suez to the east. Several studies were carried out on the Wadi Ghoweiba area including geological, hydrological, geophysical, and lithological studies (Klitzsch and Linke 1983; Salem 1988; Abdallah 1993; Abu-El-Enain et al. 1997; Sultan and Mohamed 2000; Hassan 2008; Amer et al. 2021). Recently, the northern part of EED, especially Red Sea coastal areas and El-Sokhna (which our case study is part of this area), has been considered as one of the most prominent industrial, trade, and tourism centers in Egypt. Also, highway constructions, land reclamation, and urbanization were significantly increased in the area under investigation. Thus, extensive study and accurate prediction for flash floods on Wadi Ghoweiba and surrounding areas are needed.

Therefore, the current study attempts to identify the area with sediment accumulation and erosion evolved by flash floods. Additionally, this study also focused on the new hybrid approach of ML algorithm and hydrologic indices for detecting the impacted and highly vulnerable areas. Moreover, the environmental impacts of accumulation and erosion of the sediment were investigated. The elevation thematic layer (digital elevation model (DEM)) was used to delineate the watershed, and extract the drainage network and different stream characteristics. Machine learning model (MLM) was successfully created using R environment, while the watershed characteristics were developed in GIS package. Thereafter, the hydrologic indices were calculated and identified of Wadi Ghoweiba and surrounding. Furthermore, the risk map of sediment accumulation and erosion was developed. Finally, the applied hybrid approach in the present work allows scientists and relevant flood authorities to simulate the occurrence of sediment accumulation, erosion, and the expected areas of the impending flood.

## Materials and methods

### The studied site description

**Fig. 2:**
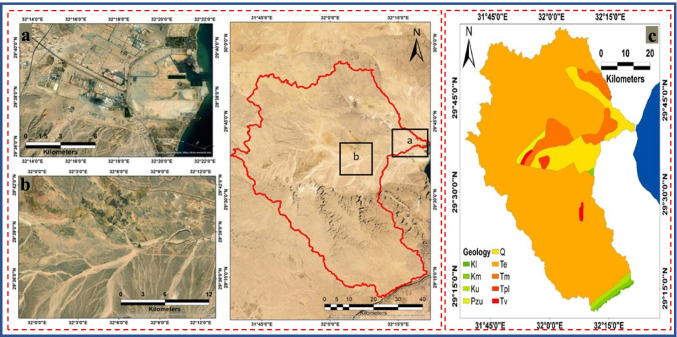
Illustrates **a** the urbanization, **b** the drainage on the study area, and **c** Lithological units of Wadi Ghoweiba (**Kl** is Lower Cretaceous, **Km** is Cenomanian and Turonian, **Ku** is Upper Cretaceous, **Pzu** is Upper Paleozoic, **Q** is Quaternary deposits, **Te** is Eocene, **Tm** is Miocene deposits, **Tpl** is Pliocene deposits, and **Tv** is Wadi Natash Volcanics)

The study area, Wadi Ghoweiba and surrounding, is located at the northern part of EED, which covers about 3258 km^2^. The Ghoweiba and surrounding are situated between 29° 10′ 56′′ N to 29° 56′ 23′′ N latitudes and 31° 38′ 54′′ to 32° 21′ 41′′ E longitudes (Fig. 1). Figure 2a,b illustrates the urbanization at the neck (a) and drainage (b) of the Wadi Ghoweiba area. The Ghoweiba basin runs directly from west to east and is surrounded by high calcareous lands from the northern and western parts (Gebel Ataqa, Gebel Kahalyia, and Gebel Abu Trifya). Moreover, the elevations inside and around the Ghoweiba basin were found to be between ~ 2 and 1247 m above sea level. Climatology, the semi-arid condition is prevailing in the area.

### Topography and geology of the studied site

**Fig. 3:**
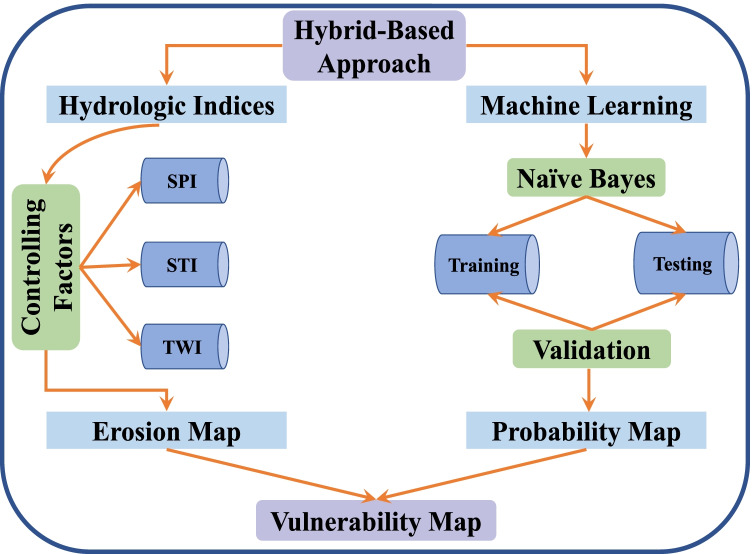
The flowchart shows the schematic diagram of the methodological framework

Geologically, various rock units were exposed and identified in the area ranging from the Jurassic to Quaternary in age. The thickness of exposed rocks in Wadi Ghoweiba and surrounding (Wadi Bada’a) are over 1100 m (Abdallah 1993). The most exposed rock units, as shown in Fig. 3, are Eocene, Oligocene, and Miocene (Hassan 2008). Variegated colors and cross-bedded sandstone along with interbeds of mudstone and siltstone represent the Jurassic age. Three rock units representing the Cretaceous age in the area under investigation including the chalky limestone, Galala, and Malha units from top to base (Fig. 2c). The Eocene rocks are represented in Wadi Ghoweiba by the Nummulitic limestones, which are found in the main part of high lands (i.e., El-Galala El-Bahariya, Gabal Ataqa, and Gabal). Two units characterized the Oligocene rocks include lower and upper units. The lower Oligocene unit is varicolored and composed of quartzites, unstratified sands, and gravels. However, the upper unit is exposed in the center of the area under investigation and comprises Gabal El Ahmer Formation basalt sheets (Fig. 2c). The Miocene succession revealed in the Sadat region is subdivided from top to base as follows: Hagul Formation (Late Miocene), Hommath Formation (Middle Miocene), and Sadat Formation (Early Miocene). Recent deposits were also found in the study region such as sands, gravels, clay, sabkha, and sediment accumulations. Structurally, the study site is highly deformed and represented by horsted block depression. Furthermore, various fault trends identified include NNW-SSE faults, E-W faults, and WNW-ESE faults (EL-Rayes et al. 2009).

### Dataset

In this study, coupling the hydrologic indices with the Naïve Bayes (NïB) method to propose a novel hybrid machine learning (HML) model was applied. Within the NïB algorithm, it is easy to predict the class of test dataset, and it is also performing well in multi-class prediction. In simple understanding, NïB classifier assumes that the presence or absence of a particular class feature is unrelated to the presence or absence of any other feature. The HML model is used for flash flood forecast purposes in areas of arid and semi-arid characteristics. An integrative approach of hydrologic indices and Bayesian technique were implemented to develop flash flood risk, sediment accumulation, and erosion assessments. All predictor variables of this study were processed in ArcGIS (10.5) and the R computing environment for the modeling step for naïve Bayes (R 3.6). A comprehensive framework developed in this study was constructed including the following steps: (i) creation and preparation of variables; (ii) creation of erosion map using stream transport index (STI), stream power index (SPI), and topographic wetness index (TWI) variables; (iii) develop the probability map by NïB approach; and finally (iv) creation of final hazard map. A schematic diagram of the methodological framework is described in Fig. 3. Table 1 represents the dataset used in the present work.

**Table 1:** Dataset used in the present work

Input data	Data Used		Output
	Type	Resolution	
Elevation	Raster	30 m	DEM, slope, curvature, aspect
Drainage network	Vector	from DEM 30 m	Stream density
Drainage network	Vector	from DEM 30 m	Distance from streams
FlowDir and Slope	Calculated	30 m	Stream power index
FlowDir and Slope	Calculated	30 m	Sediment transport index
FlowDir and Slope	Calculated	30 m	Topographic wetness index
Inventory dataset	Vector	Randomly	Training and testing

### Methods

In order to develop the current hybrid approach, hydrologic indices and NïB were calculated. NïB was used to determine the probability by utilizing Bayes’ theorem (derived from Bayesian statistics). Based on the assumption of features that are independent of class, NïB significantly simplifies learning as the following equation (Eq. (1)):

1 $${p}_{(x,C)}={\prod }_{i=1}^{n}P\left({x}_{i}|c\right)$$

where $c$ is the class and $x is \mathrm{ point out to feature vector}=\left({x}_{1},{x}_{1}, \dots \dots {x}_{n}\right)$.

**Fig. 4:**
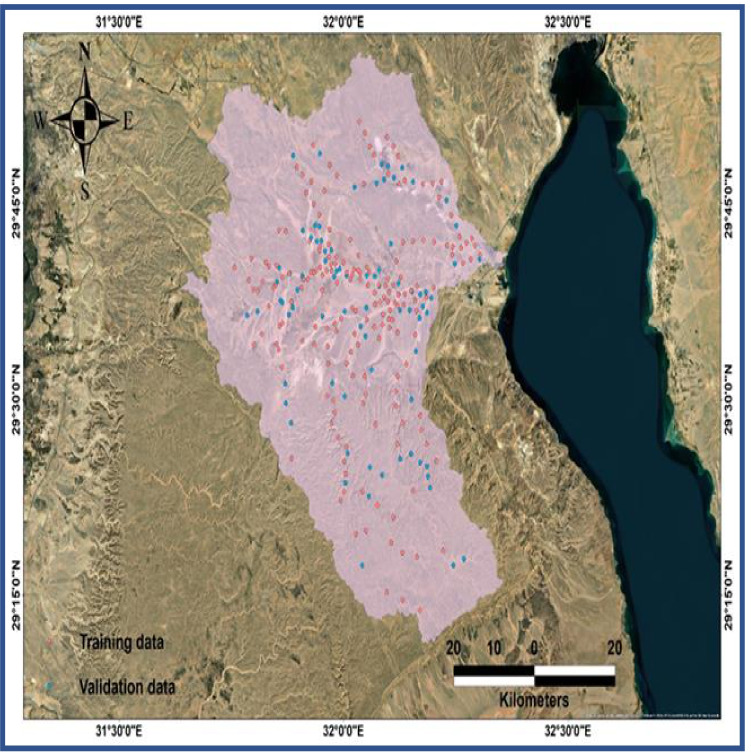
The distribution of training (pink color) and validation (blue color) datasets on the studied site

NïB approach has been previously used to determine the probability of landslides by other authors (Pham et al. 2017; Youssef and Pourghasemi 2021). In the case of classification error (zero–one loss), the uniformity of fit to a probability distribution is not always existing such as the example of the relevance of independence assumption. As an alternative, in the actual and expected distributions converge, the ideal classifier was found as a superior possible class (Abu El-Magd et al. 2021a), these findings indicate that NïB has better results even if its independence assumption is violated. In the current study, a total of 189 locations from the field survey were employed as training and validation datasets. Figure 4 illustrates the distribution of training (pink color) and validation (blue color) datasets on the studied site. The training dataset is the samples that were used to create the model. However, the testing dataset or validation dataset is the dataset that was used to qualify performance (Kuhn and Johnson 2013).

## Predictor variables

**Fig. 5:**
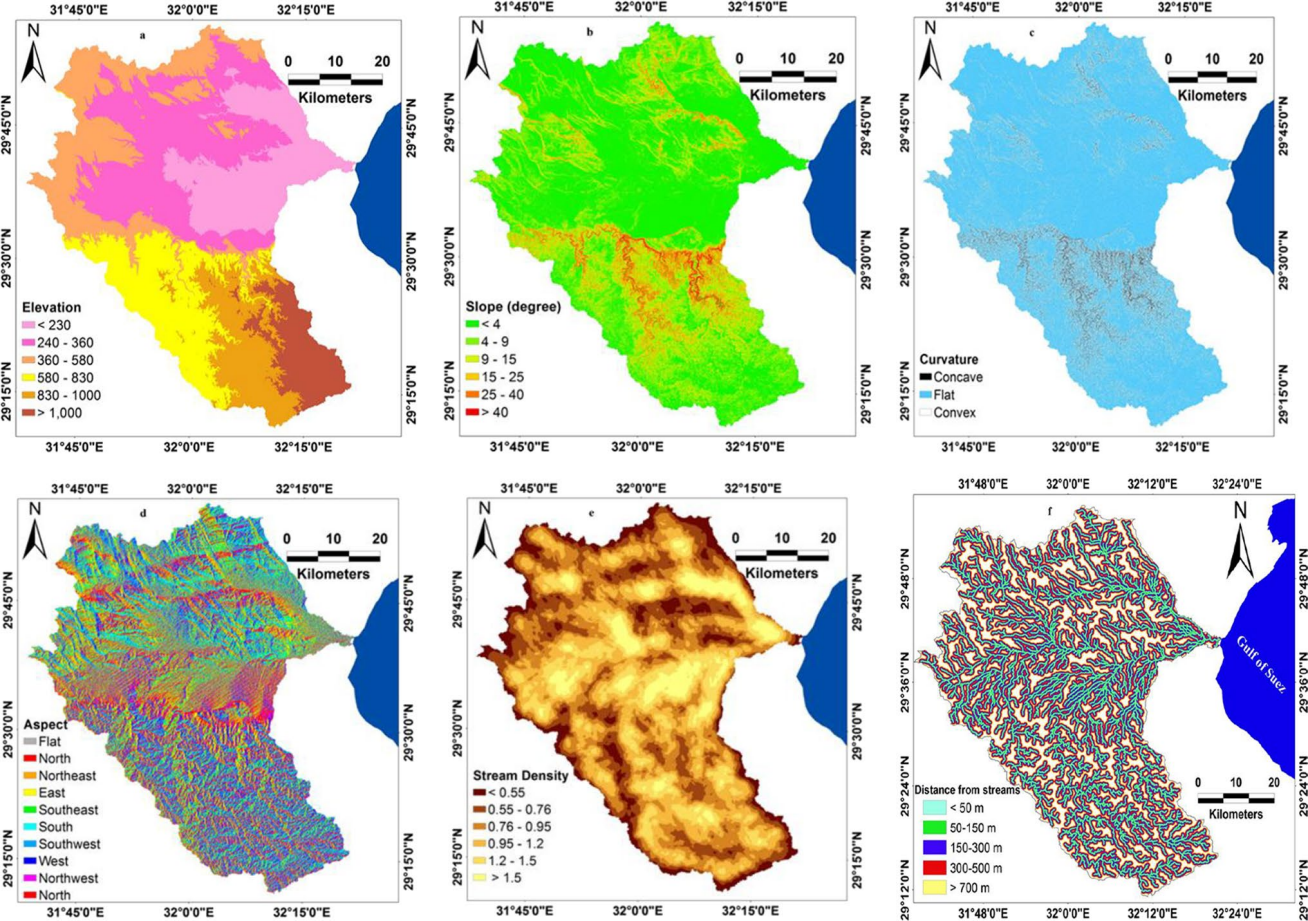
Flood controlling factors; **a** DEM, **b** slope, **c** curvature, **d** aspect, **e** stream density, and **f** distance from stream network

A variety of predictor variables for Wadi Ghoweiba and its surrounding were implemented and processed in ArcGIS (10.5). Determination of the flood controlling factors and/or predictor variables varies greatly from one watershed to another. The controlling factors were selected in susceptibility mapping depending on watershed characteristics (Sanyal and Lu 2004; Bui et al. 2016). A combination of datasets including SPI, STI, TWI, DEM, lithological units, slope angle, stream density (SD), curvature, slope aspect (SA), and distance from the streams (DS) was assigned. Watershed DEM was acquired from (http://earthexplorer.usgs.gov/) with a resolution of 30 m for the Ghoweiba basin. Several studies were reported by other authors (Cao et al. 2016; Chapi et al. 2017) confirmed that the DEM is an important and effective controlling factor for flooding events. Since the water tends to accumulate in the watershed areas of lower topography or elevation that have potential higher flooding occurrence. DEM (Fig. 5a) was classified into six classes including < 230, 230–360, 360–580, 580–830, 830–1000, and > 1000 m amsl. Moreover, one other effective controlling factor of flood event occurrence is the slope angle of a watershed (Khosravi et al. 2016; Zeng et al. 2020). It is believed that the higher slope resulted in a lower infiltration rate and consequently higher water velocity. Furthermore, the gentle slopes are more susceptible and prone to flooding that capturing a huge water quantity. Six slope intervals were constructed for slope map including < 4, 4–9, 9–15, 15–25, 25–40, and > 40 (Fig. 5b). Curvature in the present work (Fig. 5c) was categorized into three categories namely convex, concave, and flat. According to Young and Mutchler (1969), the concave class has more potential generation for runoff. Slope aspect was selected in this study (Fig. 5d), due to the aspect exhibits an impact on soil erosion and rainfall (Hurni 1989; Ragab et al. 2003). Tehrany et al. (2017) revealed that all classes of slope aspect exhibited a relationship with flooding, except flat class. Stefanidis and Stathis (2013) concluded that the geological subsoil, especially torrential petrographic formations, is a natural factor that determines flood hazard, both in terms of erodibility and permeability. SD, as stated by many researchers (Tehrany et al. 2015; Chapi et al. 2017), has an important effect on flooding events. SD was calculated in ArcMap by dividing the length of stream (m) on the basin area (km^2^) (Elmore et al. 2013). Six intervals of stream density were constructed including < 0.55, 0.55–0.76, 0.76–0.95, 0.95–1.2, 1.2–1.5, and > 1.5 (Fig. 5e). It is clear that the most impacted areas during flooding events are areas near to stream network. Therefore, distance from stream network (DSN) is an effective factor in controlling flooding events. The areas of far distance from the streams network are a lower probability of flooding occurrence. Streams network were extracted from elevation and DSN was generated within watershed with buffer zones of < 50, 50–150, 150–300, 300–500, 500–700, > 1000 (Fig. 5f).

**Fig. 6:**
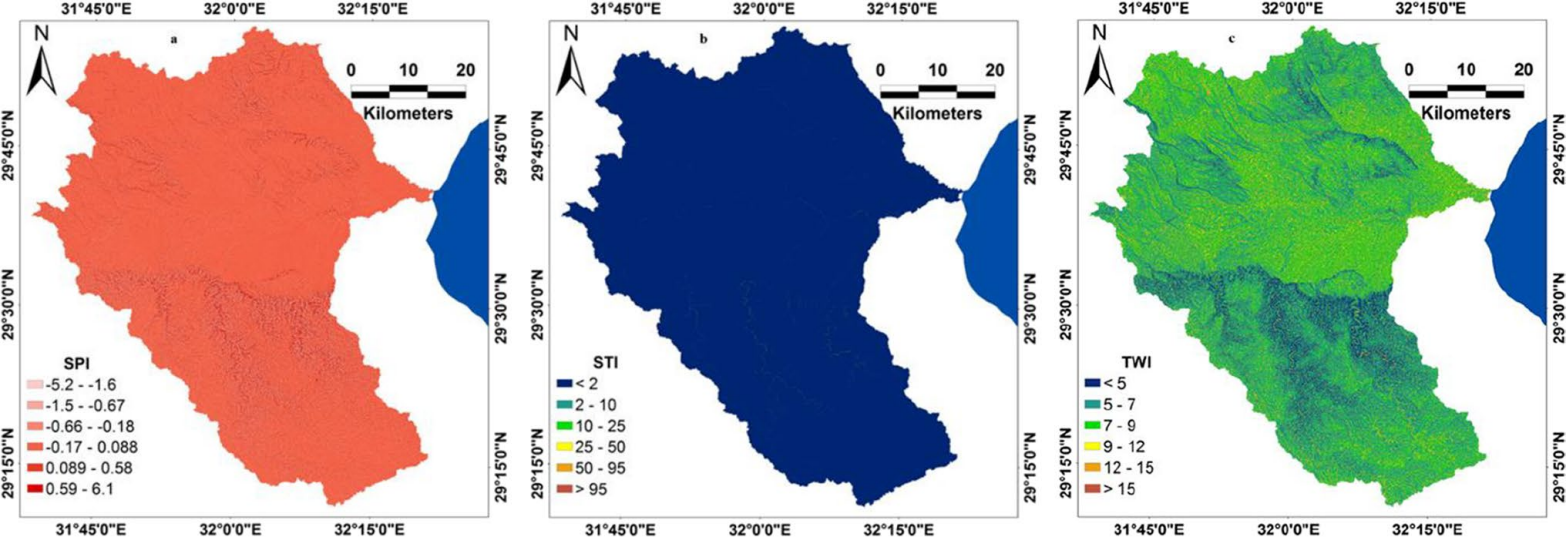
Hydrologic indices; **a** SPI, **b** STI, and **c** TWI

All predictor variables and hydrologic indices (Fig. 6a–c) were processed in ArcGIS environment. Bui et al. (2016) stated that SPI and TWI are two significant hydrologic variables that can be used to assess the flood-prone areas spatial variation. SPI is utilized to determine the erosive strength of the watershed and water discharge compared to a given region within the watershed (Poudyal et al. 2010). However, SPI is attributed to the status of soil water quality in a watershed in addition to the ability of floodwaters to drain down (Cao et al. 2016). Furthermore, the harsh strength of flooding is shown by SPI. The high SPI value indicates that the flood power is high. While, the low SPI value implies that regions in the basin have the capacity for flow accumulation (Turoğlu and Dölek 2011). STI, which refers to stream transport index, is defined based on the transport potential limiting sediment flux and catchment evolution theories of erosion. Also, STI is indicated to be a non-linear function for particular discharge and slope (Moore and Wilson 1992). Mathematically, TWI (Beven and Kirkby 1979), STI (Moore and Wilson 1992), and SPI (Cao et al. 2016) can be determined from the following equations (Eqs. (2–4));

2 $$TWI = \left(\frac{\alpha }{\mathrm{tan}\beta }\right)$$

where $\mathrm{tan}\beta$ refers to the slope angle at a specific point and $\alpha$ is the cumulative upslope drainage area through a point (per contour unit length).

3 $$\mathrm{SPI }= \left[\mathit{ln} \left({A}_{s }+0.001\right) \times \left(\left(\beta /100\right)+0.001\right)\right]$$

where ${A}_{s}$ is the accumulation of basin flow, $\beta$ is the basin slope, and “$ln$” is constant refers to the Napierian logarithm. However, the flow accumulation demonstrates regions that contribute to the overland flow.

4 $$\mathrm{STI }= \left[\left(m+1\right)\times ({A}_{s}/22.13{)}^{m}\times (\mathrm{sin}\beta /0.0896{)}^{n}\right]$$

where $$m$$ = 0.4 and $$n$$ = 1.3.

## Results and discussions

### Variable importance

**Fig. 7:**
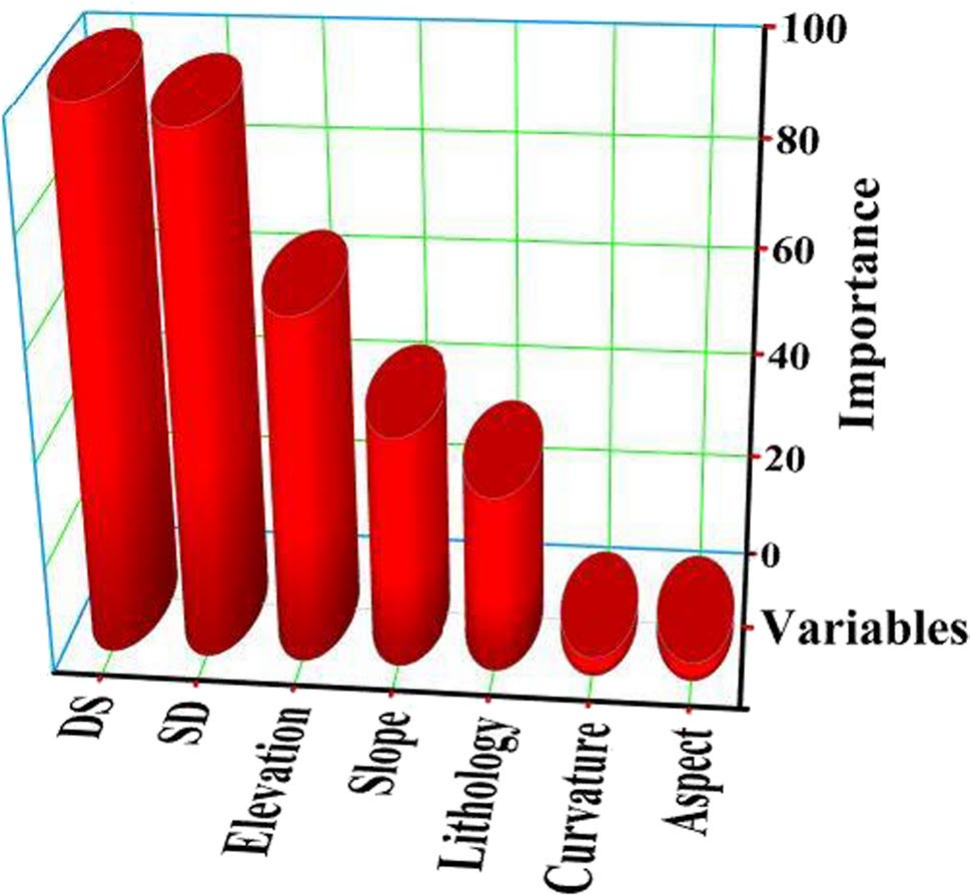
Shows the prediction importance of seven flood conditioning factors

Through time, ML techniques have evolved, focused on learning the data in order to improve the understanding of the problem (Mitchell 1997; Bhattacharya et al. 2007). Traditional models are primarily used in flood analysis and forecasting. However, these models have become less attractive tools to scientists and researchers in flooding analysis. Recently, ML approaches provided a promising technique for natural hazards prediction and forecasting. However, the potential risk of flash floods in the study site is mainly attributed to the sediment load, and water accumulation drained downstream in the east (Gulf of Suez). To understand the issues mentioned above, in the case of the Wadi Ghoweiba watershed, ten conditioning factors such as SPI, STI, and TWI were applied to generate the sediment accumulation and erosion map. Other spatial datasets of the controlling factors including curvature, slope, slope aspect, elevation, SD, DSN, and lithology were used to construct the ML model. ML approaches and hybrid-based methods have been utilized to perform more accurate analysis, forecasting and prediction compared to the conventional methods (Pham et al. 2016, 2017). Several steps were applied in this study to prepare and extract various datasets from different resources. Extensive field surveys and data collection from multiple sources were carried out to construct the inventory map of the obtained model. Nearly about 189 locations of sediment accumulation and erosions were identified, which were classified randomly to 70% training dataset and the remained locations (30%) were used for testing purposes. However, selecting the variables is an essential step in model building. Therefore, determination of the effect of controlling factors and their predictive power were conducted via R using variable importance. The variable’s importance was also calculated, indicating that the higher importance value was the strongly conditioning factor affecting the model. In the present study, a hybrid-based model was developed by combining NïB approach and hydrologic indices for flash flood analysis and prediction. The new hybrid model’s performance was compared with a standalone NïB algorithm performance. The results indicated that distance from stream network followed by stream density was the most critical flood conditioning factor in the area. Several researchers have concluded that the stream density and slope (Abu El-Magd et al. 2021a) or land use (Costache et al. 2020; Ali et al. 2020) are significantly correlated with flood potentiality; based on the study site and hydro-climatological conditions. However, the current study proved the less contribution of topography factors in the flood susceptibility. These factors played a significant role in the occurrence of flood inundations. Consequently, impervious surfaces such as roads and buildings decrease the infiltration capacity while simultaneously increasing surface runoff, which can significantly increase total runoff. Figure 7 illustrates the prediction importance of seven flood conditioning factors. The importance figure indicates that the aspect and curvature have the lowest importance effects on the model with a value of 3.44 and 3.45%, respectively. However, the distance to the stream network (100%) and stream density (96%) showed the higher predictive power for the obtained model. Meanwhile, the other factors such as elevation, slope, and lithology were moderately affected the model prediction percentage with values ranging from 33% up to 64% (Fig. 7). This supports the fact that flooding and sediment accumulation occurs in flat and gentle slope areas.

**Fig. 8:**
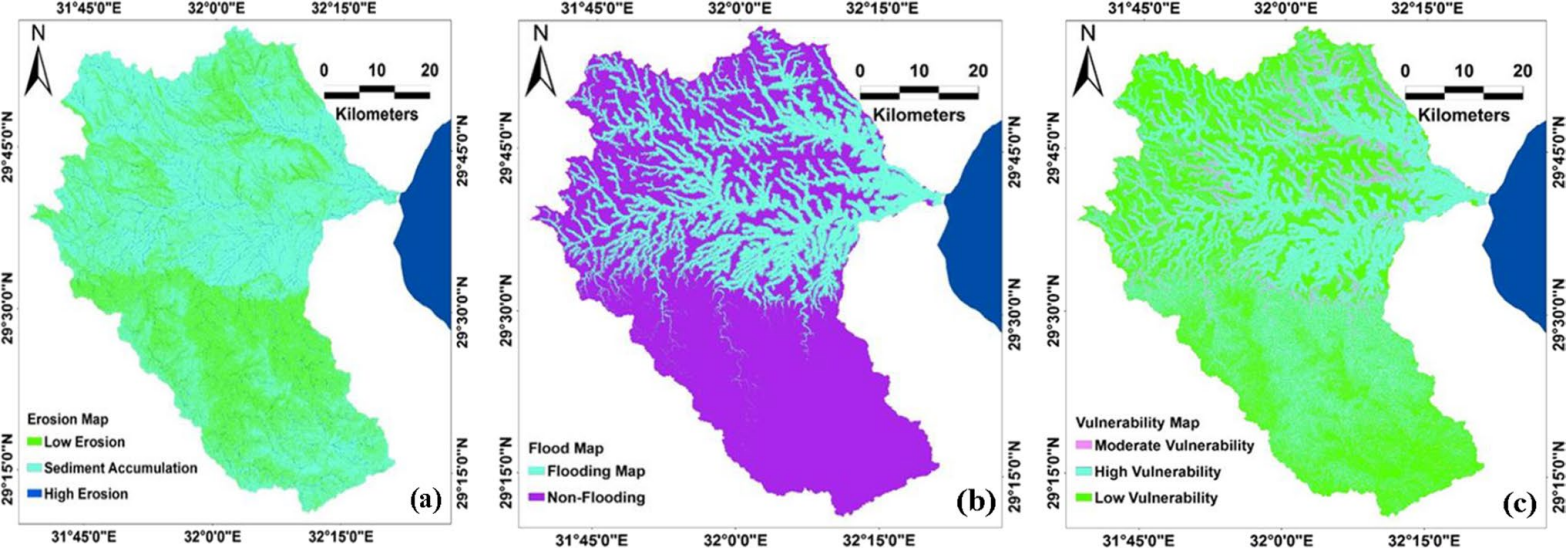
Shows **a** erosion map, **b** flooding/non-flooding study area prediction map, and **c** vulnerability map

Moreover, the current study aimed to analyze and discuss the prediction of sediment accumulation in the study site. The results have shown that sediment accumulation is linked to low topography and gentle slope (Fig. 8a). A significant contribution of the streams network is associated with sediment erosion (high erosion) and the foot of high lands. Indeed, the vegetation cover and rock type in arid land are factors that could reduce the erosion rate. Furthermore, the flash flood water velocity process is responsible for the sediment accumulation and erosion rate.

### Flooding and non-flooding areas

The areas of Red Sea coast and especially the identified for this study have a long history of flooding events and are regularly affected by flooding. According to the results and the interpretation of generated flood susceptibility map (Fig. 8b), the areas closer to the stream network are the most affected parts. Consequently, areas of the distance between 0 m to less than 300 m from the streams network would be impacted by flash floods. It could be concluded that the areas in the east associated with gentle slopes contribute to the flooding events. Furthermore, the northern and middle parts of the Goweiba basin are more associated with the flash flood than the southern part. Table 2 demonstrates the surface area of flooding and non-flooding. Based on the flooding and non-flooding generated prediction map study area, the flooding area was found to be 1013.25 km^2^ compared to 2244.29 km^2^ of the non-flooding area.

**Table 2:** Surface area of flooding and non-flooding regions

Category	Flooding area	Non-flooding area
Area (Km^2^)	1013.25	2244.29

### Vulnerability map

The originality of this study and its practical value lies in providing an effective hybrid approach for flash flood susceptibility analysis and prediction. It is obviously clear that the possibility of improving the prediction accuracy for ML algorithms by combining or hybrid approaches. Since the generated vulnerability map (Fig. 8c) exhibited better accuracy than the standalone ML model, the flood susceptibility map was classified into high, moderate, and low vulnerability. The high vulnerability is mostly located in the northern and middle areas of the basin. Some patches of high vulnerability were observed in the southern part of Wadi Ghoweiba.

### Model validation

**Fig. 9:**
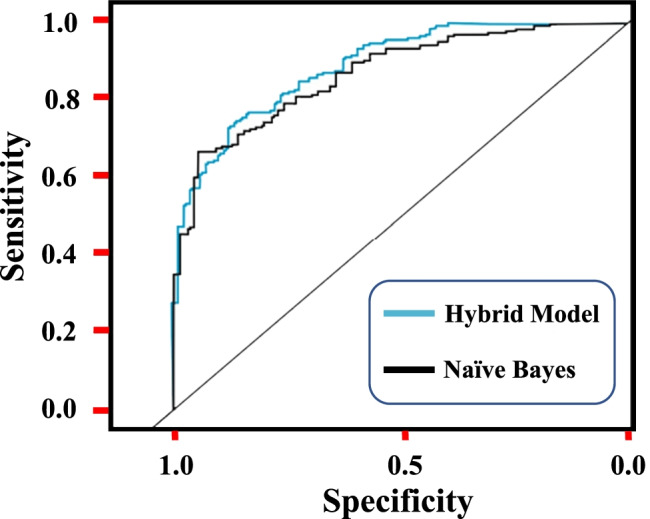
Shows the accuracy for the new hybrid model and Naïve Bayes prediction of the studied site

Generally, model validation quantifies the performance that could be expected from a given MLM on unseen data. Figure 9 illustrates the performance of the obtained model utilizing the training and testing dataset. In this model, the receiver operating characteristic (ROC) and area under the curve (AUC) for testing and training datasets were applied to evaluate the obtained hybrid model accuracy compared to naïve Bayes model. During the modeling, one of the first considerations is the selection of the utilized samples in order to evaluate the performance. In an ideal case, in order to get an accurate assessment of the model, the used samples should not be previously processed (developed or fine-tuned). Toward improving prediction and accuracy, this work presents a hybrid model for flash flood and sediment accumulation analysis and prediction. The model performance shows that the accuracy is 87.7% and 90.8% for naïve Bayes and the new hybrid approach, respectively.

### Environmental impact

Hundreds of individuals lost their lives and severe damages were recorded worldwide yearly due to flood consequences. Floods create massive disruptions that affect infrastructures, power lines, and industry. Associated social and environmental impacts of flash floods in the study area could limit the development and landscaping. Predicting and delineating flood-prone areas is a crucial element for any flood alleviation strategy. Predicting flash floods and flood-prone regions dramatically impacts the environment and humans. First, the studied site extended to the Gulf of Suez coast, which is an important location for industrial and touristic activities. Second, the study reveals the distribution and accumulation of sediments over large areas, resulting from flood erosions. The obtained information for the sediment erosion is also helpful to replenish valuable topsoil for agricultural activities in the basin. The flash flood water could be partially leaked into raw sewage causing a problematic disruption and blockage in the drainage systems in the coastal cities. Therefore, the present work attempts to identify the potential zones of a flash flood, sediment erosion, and accumulation for further development.

## Conclusion

A newly proposed hybrid technique for assessing flood accumulation and erosion in the present work by combining hydrologic indices and NïB was successfully applied. Results of the implemented hybrid-based approach showed that an increase in the accuracy of NïB model was achieved. Consequently, the obtained hybrid-based approach can be used as an improved alternative approach to developing the simulations and forecasting of flood hazards and sediment accumulation. Various thematic layers were used as controlling factors for model inputs. The controlling factors of the obtained model were selected based on the literature and flood affecting parameters. Training and testing datasets (189 locations) from the field survey were utilized for training and testing the model. The NïB approach exhibited a model accuracy of 87.7%, while the training and testing datasets were classified randomly into 70 and 30%, respectively. However, the applied hybrid-based method in the current work demonstrated a significantly higher accuracy reaches to 90.8%. The proposed model performs well for spatial analysis and prediction for flood sediment accumulation and erosions. The limitation in the obtained hybrid model was the hydraulic data such as the velocity, sediment load, flood inundation, etc. The output maps and thematic layers can help the planner and authorities in semi-arid regions in flood management and unplanned urbanization.

## Data Availability

The datasets used and/or analyzed during the current study are available from the corresponding author on reasonable request.
